# The impact of tumor burden at the initial hepatectomy on the recurrence-to-death survival after repeat surgical resection/radiofrequency ablation: a retrospective study

**DOI:** 10.1186/s12893-022-01643-7

**Published:** 2022-05-18

**Authors:** Youwei Wu, Wei Peng, Junyi Shen, Xiaoyun Zhang, Chuan Li, Tianfu Wen

**Affiliations:** Department of Liver Surgery & Liver Transplantation Center, West China Hospital, Sichuan University, Chengdu, 610041 Sichuan China

**Keywords:** Hepatocellular carcinoma, Recurrence, Milan criteria, Radiofrequency ablation, Surgical resection

## Abstract

**Background:**

Previous studies have reported the surgical resection (SR) and radiofrequency ablation (RFA) could achieve comparable recurrence-to-death survival (RTDS). However, the impact of primary tumor burden on RTDS of patients with recurrent hepatocellular carcinoma (HCC) following SR or RFA has not been clarified.

**Methods:**

From January 2009 to March 2015, 171 patients who underwent initial hepatectomy and second curative treatments in West China Hospital were retrospectively analyzed. Survival analysis was performed by the Kaplan–Meier method. Risk factors were identified using the Cox proportional hazard model.

**Results:**

At initial hepatectomy, 96 patients (56.1%) were diagnosed with HCC within the Milan criteria (MC), and 75 patients (43.9%) were HCC beyond the MC. The clinicopathological features and re-treatment methods of recurrent HCC were similar between patients with primary HCC within or beyond the MC. Patients with primary HCC within the MC had longer recurrence time (31.4 ± 24.2 months vs. 20.2 ± 16 months, P < 0.001). The 1- and 3- year RTDS within and beyond the MC group were 88.8%, 57.6% and 79.0%, 46.3%, respectively (P = 0.093). In multivariate analysis, the recurrence time, tumor size and AFP > 400 ng/mL at the time of recurrence were associated with RTDS.

**Conclusions:**

The primary tumor burden had no impact on RTDS, but had an impact on recurrence time. The recurrence time had an impact on RTDS and might be a good index to reflect the biology of recurrent HCC.

## Background

Hepatocellular carcinoma (HCC) is one of the leading causes of cancer-related mortality worldwide [1]. The potential curative treatment modalities for HCC include liver transplantation, surgical resection (SR), and radiofrequency ablation (RFA). In clinical practice, many HCC patients present with intermediate or advanced-stage disease at diagnosis because of the lack of early-stage symptoms. Multiple evidences including one from a randomized controlled trial (RCT) have suggested that surgery could provide better survival than other treatments [2, 3]. However, the long-term prognosis of HCC remains unsatisfactory given the high frequency of tumor recurrence (5-year recurrence rate, ~ 70%) [4]. Therefore, most patients required subsequent treatments, such as repeat surgical resection (RSR), RFA, or transcatheter arterial chemoembolization (TACE). Multicentric occurrence (MO) and intrahepatic metastasis (IM) are two types of recurrence with different prognosis [5]. However, it is difficult to define the clonal origin of recurrent HCC limited by the current pathological examination. Therefore, in the current study, we analyzed the postoperative recurrence survival based on the initial and recurrent tumor stage. For treatment modalities, numerous studies, including one meta-analysis, suggested both RSR and RFA are comparable in terms of long-term survival for patients with recurrent HCC. Further, a recent RCT also reported no statistically significant difference in survival outcomes after RSR versus RFA for patients with early-stage recurrent HCC [6–9]. It is now accepted that for those with early-stage or locally recurred HCC, RSR or RFA is the first line of treatment. In the current study, we included patients who underwent RSR or RFA or both curative methods. Previous studies showed the 5-year survival rate of patients beyond the Milan criteria (MC) could achieve 39–60%, lower than that for patients within the MC [3, 10–12]. For those with comparable tumor stage at recurrence time, the effect of primary HCC within or beyond the MC on the recurrence-to-death survival (RTDS) remains unclear after curative treatments.

A previous study showed that 30–60% of recurrent or metastatic tumors harbor clones different from the primary tumor, suggesting differences in terms of biology and prognosis from the primary HCC [13]. The prognostic factors associated with post recurrence survival should be further investigated.

In this study, we aimed to investigate the impact of primary HCC within or beyond the MC at the initial hepatectomy on the RTDS after curative treatments in patients with local or regional recurrent HCC. The prognostic factors associated with RTDS were also analyzed.

## Methods

### Study patients

Between January 2009 and March 2015, 171 patients who underwent hepatectomy for primary HCC and RSR or RFA for recurrent HCC in the Department of Liver Surgery & Liver Transplantation Centre at our institute were enrolled for this study. Curative treatment was defined as negative surgical margin, or no residual tumors detected by computed tomography (CT) and/or magnetic resonance imaging (MRI) within one month. All patients were followed-up until November 2017 or until death. The inclusion criteria were as follows: patients (1) initially treated by hepatectomy; (2) with the liver function was classified as Child–Pugh A, and (3) with the first local or regional relapses of HCC treated by resection or RFA or both curative methods. The exclusion criteria were: patients (1) with the major vascular invasion at the first treatment; (2) with diffuse or distant metastasis at the first treatment; (3) who underwent salvage liver transplantation; and (4) with incomplete clinicopathological or follow-up data. In the current study, there were 10 cases with regional relapses of HCC (extrahepatic metastasis). They were recommended resection by a multidisciplinary team in West China Hospital. The study was approved by the Ethics Committee on Biomedical Research, West China Hospital of Sichuan University (No.2017062) and was conducted under the tenets of the Declaration of Helsinki. The work has been reported in line with the STROCSS criteria [14].

At the first surgery, clinicopathological variables including age, sex, underlying liver disease, and preoperative laboratory results such as alpha-fetoprotein (AFP), tumor number, largest tumor diameter, tumor differentiation, microvascular invasion (MVI), satellite lesions, and liver cirrhosis were recorded. At the second surgery, clinicopathological variables including preoperative laboratory results such as AFP; and levels of aminotransferase (ALT), aspartate transaminase (AST), and total bilirubin (TBIL); tumor number; and largest tumor diameter were collected [15, 16]. At the initial hepatectomy, 95 patients were within the Milan criteria (a single tumor < 5 cm or with up to three nodules < 3 cm) and 76 patients were beyond the Milan criteria.

### Follow-up

All patients were followed-up in the first postoperative month, then every 3 months for the first 2 years, and 6 months thereafter until the death of the patient. Before and after the operation, antiviral drugs were administered to the patients according to the guidelines. Blood routine; liver function tests; AFP levels; HBV markers; HBV-DNA levels; and imaging examinations such as liver ultrasonography, CT, or MRI were included at each investigation. Tumor recurrence was identified based on either at least two positive radiological examinations or one positive radiological examination and increased AFP levels, including the intrahepatic and extrahepatic metastasis. The recurrence-free survival (RFS) time was defined as the interval between the operation and the first incidence of detectable recurrence (intrahepatic or extrahepatic metastasis). The overall survival (OS) time was defined as the interval between the operation and death or the last follow-up. The RTDS time was defined as the interval between HCC recurrence and death or the last follow-up. The last follow-up date was at the end of November 2017 or until death.

### Statistical analysis

Categorical variables were summarized using frequency and percentage and were compared using Fisher’s exact test. Continuous variables were expressed as mean ± standard deviation and were compared using the Student’s *t-*test or Mann–Whitney *U* test (for non-normally distributed data). The overall survival (OS) and RTDS were estimated using Kaplan–Meier survival curve plots and were compared using the log-rank test. Multivariate analyses of post recurrence survival were tested using the Cox proportional hazards model. Potential risk factors with P < 0.1 in the univariate analysis were included in the multivariate analysis model using forward step-wise selection process [17]. A *P* value < 0.05 was considered statistically significant. The analysis was performed using the SPSS20.0 software (IBM Corp, Armonk, NY).

## Results

### Clinicopathological characteristics

There were 171 patients in the current study. At initial hepatectomy, 96 patients (56.1%) were diagnosed with HCC within MC, and 75 patients (43.9%) were HCC beyond MC. The clinicopathological data are shown in Table 1. At the initial stage, the average tumor size of patients within and beyond the MC group was 3.3 ± 1.1 cm and 7.9 ± 3.2 cm, respectively (P < 0.001). Furthermore, there were 83 (86.5%) and 55 (73.3%) patients with one tumor, 10 (10.4%) and 16 (21.3%) patients with two tumors, and 3 (3.1%) and 4 (5.3%) patients with at least three tumors (*P* = 0.095), respectively, in the within and beyond MC groups. Patients in the beyond MC group had a higher rate of microvascular invasion than those in the within MC group (38.7 vs. 19.8%) (*P* = 0.01). However, those in the within MC group showed a higher rate of liver cirrhosis than those in the beyond MC group (79.1 vs. 61.3%, *P* = 0.036). And patients with HCC within the Milan criteria had a longer time to recurrence than those with HCC beyond the Milan criteria (31.4 ± 24.2 months vs. 20.2 ± 16 months, *P* < 0.001). There were no significant intergroup differences with respect to age, sex, HBV infection, tumor differentiation, rate of satellite lesions, and AFP > 400 ng/mL. At the time of recurrence, the average tumor size was 3.2 ± 2.4 cm and 3.2 ± 2.1 cm, respectively (*P* = 0.845). There were 75 (78.1%) and 52 (69.3%) patients with one tumor, 10 (10.4%) and 11 (11.4%) patients with two tumors, and 11 (11.5%) and 12 (16.0%) patients with at least three tumors (*P* = 0.445), respectively, in the within and beyond MC groups. patients with primary HCC beyond MC seemed to have more frequent occurrences of extrahepatic invasion, although this was not statistically significant. The other variables were comparable between both groups (Table 1). Patients with primary HCC beyond MC or with MVI had a shorter time to recurrence (Table 2).

**Table 1:** Demographic and clinical data at initial hepatectomy and at recurrence time

	Within Milan criteria	Beyond Milan criteria	OR	95% CI	p value
	(n = 96)	(n = 75)			
At initial hepatectomy					
Age (years old)	49.6 ± 11.9	48.5 ± 12.5			0.548
Gender(male/female)	77/19	Jul-68			0.085
Positive HBsAg	84 (87.5)	67 (89.3)			0.813
HBV-DNA > 103	22 (29.3)	26 (27.1)			0.864
Tumor size (cm)	3.3 ± 1.1	7.9 ± 3.2			0.001*
Tumor number					0.095
One	83 (86.5)	55 (73.3)			
Two	10 (10.4)	16 (21.3)			
More	3 (3.1)	4 (5.3)			
Differentiation(poor/moderate-well)	34/62	27/48			0.52
Microvascular invasion	19 (19.8)	29 (38.7)	2.555	1.289–5.064	0.010*
Satellite lesions	9 (9.4)	9 (12.0)			0.622
Liver cirrhosis	76 (79.1)	46 (61.3)	2.396	1.217–4.715	0.036*
AFP(> 400 ng/ml)	30 (31.2)	27 (36.0)			0.519
Recurrence time(mo)	31.4 ± 24.2	20.2 ± 16.1			< 0.001*
Treatments after recurrence					
Resection	48 (50.0)	32 (42.7)			0.552
RFA	44 (45.8)	38 (50.7)			
Resection + RFA	4 (4.2)	5 (6.7)			
At recurrence time					
Tumor size (cm)	3.2 ± 2.4	3.2 ± 2.1			0.845
Tumor number					0.445
One	75 (78.1)	52 (69.3)			
Two	10 (10.4)	11 (14.7)			
More	11 (11.5)	12 (16)			
Satellite lesions	2 (2.1)	1 (1.3)			1
Macrovascular invasion	1 (1.0)	1 (1.3)			1
Extrahepatic invasion	3 (3.1)	7 (9.3)			0.107
Spleen	1(1.0)	0			0.101
Greater omentum	0	1 (1.3)			0.93
Abdominal wall	1 (1.0)	1 (1.3)			0.308
Diaphragm	1 (1.0)	1 (1.3)			0.198
Abdominal cavity	0	2 (2.7)			0.355
Colon	0	1 (1.3)			0.757
Lung	0	1 (1.3)			0.266
AFP(> 400 ng/ml)	12 (12.5)	17 (22.7)			0.101
TBIL(mmol/L)	15.4 ± 5.9	15.5 ± 6.8			0.93
ALT(U/L)	35.2 ± 26.3	41.8 ± 56.3			0.308
AST(U/L)	33.9 ± 17.5	38.5 ± 27.8			0.198
ALB(g/L)	42.9 ± 3.7	42.4 ± 4.0			0.355
PLR	83.7 ± 52.4	86.2 ± 51.4			0.758
NLR	2.2 ± 1.1	2.4 ± 1.5			0.266

*indicated statistically significant

*HBsAg* hepatitis B surface antigen, *HBV* hepatis B viral, *AFP* alpha fetoprotein, *TBIL* total bilirubin, *ALT* aminotransferase, *AST* aspartate transaminase, *ALB* albumin, *PLR* platelets-to-lymphocyte ratio, *NLR* neutrophil-to-Lymphocyte Ratio

**Table 2:** Comparison of recurrence time in primary tumor burden and microvascular invasion

	Beyond the Milan criteria	Within the Milan criteria	p value
Recurrence time(mo)	20.2 ± 16.1	31.4 ± 24.3	0.001
	Microvascular invasion ( +)	Microvascular invasion (−)	p value
Recurrence time(mo)	20.1 ± 21.2	29.0 ± 21.5	0.014

### Survival analysis

The follow-up period in the within and beyond MC groups was 59.6 ± 27.8 and 44.9 ± 24.2 months respectively. All patients received curative resection at the first surgery. At the time of censoring, 37 (44.4%) patients within MC and 39 (52.0%) patients beyond MC died (P = 0.089). With respect to the recurrences, 44 (45.8%) and 32 (42.7%) patients received RSR, 48 (50.0%) and 38 (50.7%) patients received RFA, and 4 (4.2%) and 5 (6.7%) patients received combined therapy, respectively, in the within and beyond MC groups (*P* = 0.552).

**Fig. 1:**
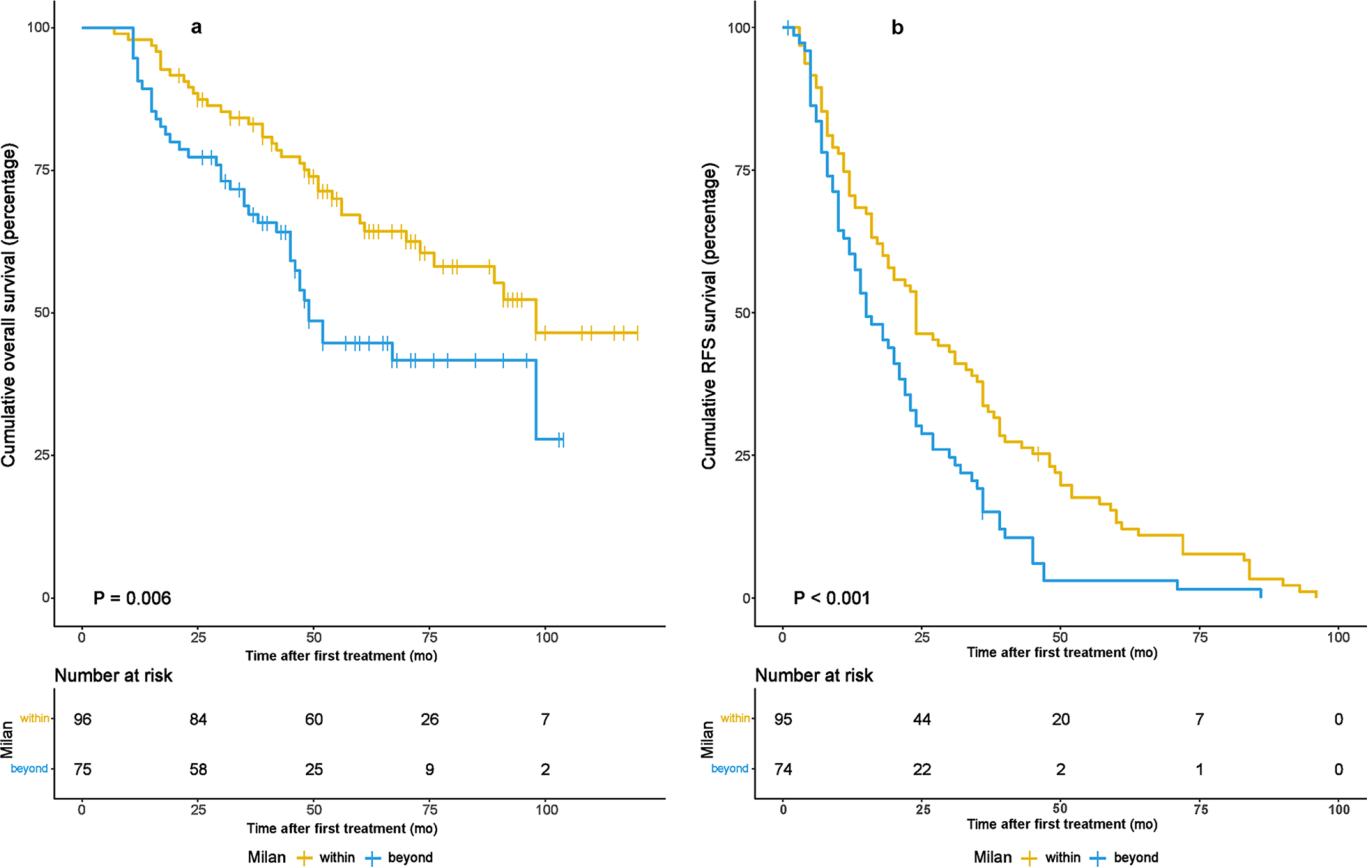
Overall survival (OS) rate **a** and recurrence-free survival (RFS) rate **b** of patients with primary HCC within and beyond MC

**Fig. 2:**
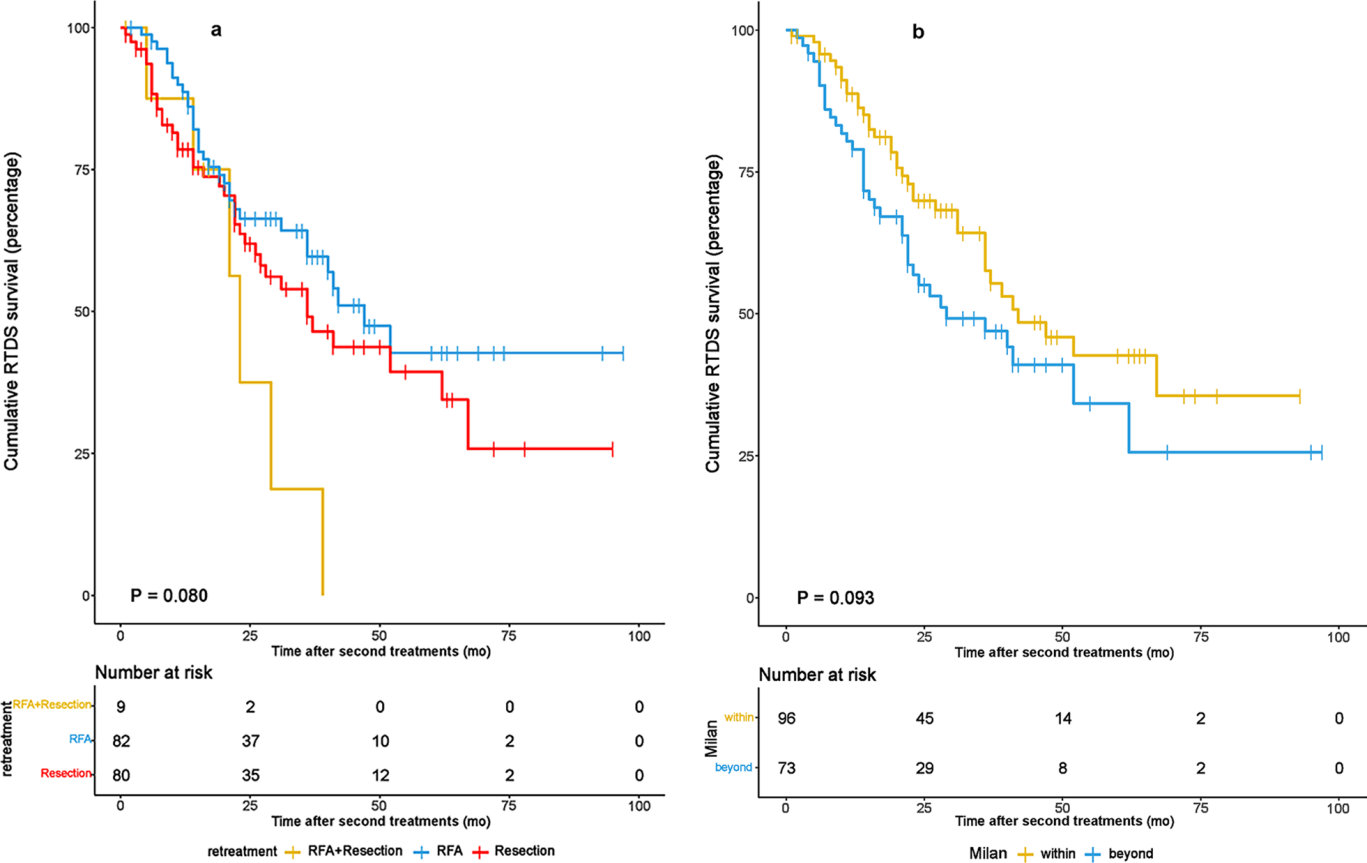
**a** Overall survival (OS) rates of the RSR/RFA/RSR + RFA group. The OS rate among the three subgroups was comparable. **b** The recurrence-to-death survival (RTDS) of patients with primary HCC within and beyond MC

The 1-, 3-, and 5-year OS rates in patients within the MC group were 96.9, 83.1, and 65.7%, respectively, and 90.7, 67.3 and 41.7%, respectively, in patients beyond the MC group. The survival curve for patients within the MC group was significantly better than that for patients beyond the MC group (Fig. 1a,  *P*= 0.006). Similarly, the 1, 3, and 5 year RFS rates were better for HCC patients within the MC group than for those with HCC beyond MC (Fig. 1b). All patients with recurrent HCC received RFA and/or resection. The RTDS survival curves were not statistically significant when all patients were stratified by treatment methods (RFA vs. resection vs. RFA + resection) (Fig. 2a). The 1 and 3 year RTDS in patients with primary HCC within the MC group was 88.8 and 57.6%, respectively, and 79.0 and 46.3%, respectively, in patients with primary HCC beyond the MC group. The survival curve for patients within the MC group was slightly better than that for patients beyond the MC group (Fig. 2b), but this difference was not significant (*P* = 0.093).

### Prognostic factors associated with RTDS

As shown in Table 3, the univariate analysis suggested that MVI, recurrence time, tumor size, tumor number, extra-hepatic metastasis, AFP > 400 ng/mL, and PLR were significant factors. Multivariate analysis showed that recurrence time (*P* = 0.009, hazard ratio [HR]: 0.978, 95% confidence interval [CI:] 0.962–0.995); tumor size (*P* = 0.001, HR: 1.149, 95% CI: 1.062–1.242); and AFP (> 400 ng/mL) (*P* < 0.001, HR: 2.465, 95% CI: 1.486–4.089) were prognostic factors after curative treatment. Tumor size and tumor number at the first surgery were not demonstrated to be related to RTDS (*P* > 0.05).

**Table 3:** Factors affecting recurrence-to-death survival (RTDS)

	Univariate analysis			Multivariate analysis		
Variable	p value	HR	95% CI	p value	HR	95% CI
At initial hepatectomy						
Age (years old)	0.355					
Gender(male/female)	0.449					
Positive HBsAg	0.129					
Positive HBeAg	0.402					
HBV-DNA	0.037	1.642	1.030–2.620			
Beyond Milan criteria	0.098	1.467	0.932–2.310			
Tumor size (cm)	0.068	1.056	0.996–1.119			
Tumor number	0.784					
Differentiation(poor/moderate-well)	0.406					
Microvascular invasion	0.015	1.787	1.117–2.860			
Satellite lesions	0.390					
Liver cirrhosis	0.676					
AFP(> 400 ng/ml)	0.595					
Recurrence time	0.003	0.975	0.959–0.991	0.009	0.978	0.962–0.995
Treatments after recurrence	0.977	0.994	0.664–1.488			
At recurrence time						
Tumor size (cm)	0.002	1.130	1.046–1.222	0.001	1.149	1.062–1.242
Tumor number	0.016	1.426	1.067–1.905			
Satellite lesions	0.474					
Macrovascular invasion	0.003	3.367	1.529–7.415			
Extrahepatic metastasis	0.016	2.363	1.174–4.758			
AFP(> 400 ng/ml)	< 0.001	2.706	1.652–4.433	< 0.001	2.465	1.486–4.089
TBIL(µmol/L)	0.444					
ALT(U/L)	0.660					
AST(U/L)	0.728					
Albumin(g/L)	0.871					
PLR	0.016	1.005	1.001–1.009			
NLR	0.692					

*HBsAg* hepatitis B surface antigen, *HBV* hepatis B viral, *AFP* alpha fetoprotein, *TBIL* total bilirubin, *ALT* aminotransferase, *AST* aspartate transaminase, *ALB* albumin, *PLR* platelets-to-lymphocyte ratio, *NLR* neutrophil-to-Lymphocyte Ratio

## Discussion

Repeat surgical resection and radiofrequency ablation are two of the most common curative therapies for recurrent HCC with acceptable long-term survival, given that liver transplantation is typically limited by organ shortage [18–21]. Consistent with previous studies, our study confirmed that the curative treatments (RSR, RFA, RFA + RSR) had no statistically significant difference in post-recurrence survival [7, 20, 22]. Despite this, we observed a tendency toward a relatively poor prognosis for patients treated using combined therapy, most likely owing to the advanced tumor stages. For the recurrent tumor, the average tumor size was about 3.2 cm. Complete tumor ablation could be inducted by RFA. In our study, the RTDS was comparable based on different treatments regardless of the primary HCC within or beyond MC. Patients with HCC beyond MC had poorer OS than those with HCC within MC (5 year OS rate: 44.7 vs. 65.7%, P = 0.001). Increased tumor size and multiple tumors negatively impacted the prognosis [23]. As reported in the study, the treatment methods, tumor number, and tumor size at recurrence between both groups showed no significant differences. Interestingly, patients with primary HCC within and beyond MC had comparable RTDS after curative treatments (5 year OS rate: 42.6% vs. 34.2%, P = 0.093). Patients with primary HCC beyond MC showed a trend for poor RTDS after curative treatments, but this association was not statistically significant. Moreover, tumor number, tumor size, or tumor beyond MC was not demonstrated to be significant prognostic factors of RTDS in the univariate and multivariate analysis. Interestingly, time to recurrence was identified as an important predictor of RTDS, which might suggest it was a better index than the primary tumor burden to represent tumor biology. This was in accordance with previous studies [6, 24]. Notably, patients with HCC beyond MC had a shorter time to recurrence than those with HCC within MC (20.2 ± 16.1 months vs. 31.4 ± 24.2 months). Features of tumor burden such as increased tumor size and multiple tumors increased the risk of recurrence [2, 25]. A short recurrence time could indicate poor tumor biology [26]. Zheng et al. demonstrated the time interval to HCC recurrence could predict the prognosis after salvage liver transplantation.[27] MVI representing the biological behavior of HCC was another significant variable predicting tumor recurrence [28, 29]. A previous study suggested that the status of MVI could be used as selection criteria for the best treatment strategy for intrahepatic recurrence [30]. However, in the current study, MVI was significant in the univariate analysis but not in the multivariate analysis for RTDS. Although the primary tumor burden or MVI is not associated with RTDS, it was closely related to recurrence time, which indirectly impacted the RTDS. We believe that the time to recurrence was a better reflector of tumor biology than the primary tumor burden.

Consistent with previous studies, the AFP level at recurrence was a prognostic factor of RTDS [31–33]. In the current study, we identified AFP > 400 ng/mL at recurrence had a negative impact on RTDS. High AFP levels were associated with highly aggressive cancer and correlated with poor prognosis [34–36]. The tumor size at recurrence had a negative impact on the prognosis because the increased tumor size was correlated with unfavorable pathological factors [37, 38]. Multiple tumors and increased tumor size are well-established risk factors for recurrence [2, 39, 40]. It cannot be denied that patients with primary HCC beyond MC might more easily develop multiple or diffuse tumor recurrence, precluding further curative treatments. Overall, patients with HCC beyond MC had a poorer prognosis than those with primary HCC within MC. However, those with early-stage HCCs and suited for curative treatments might achieve comparable RTDS.

Our study has some limitations. First, the study population in both groups was relatively small, and the follow-up period was not long enough. Second, patients in the current study might have been chosen rather carefully because many patients had advanced recurrent HCCs, especially those with HCC beyond MC. Third, the diagnosis of recurrent HCCs in RSR patients was confirmed by pathology, while the diagnosis in RFA patients was confirmed by two imaging studies combined with AFP levels. Fourth, our study only included the first treatment of patients with recurrent HCC after initial hepatectomy. Therefore, we did not collect detailed data on multiple treatments in patients with recurrent HCC.

## Conclusions

This study proved that the primary tumor burden had no impact on RTDS, but had an impact on the recurrence time. The recurrence time might be a good parameter to represent the biology of the recurrent HCC. Patients should be subjected to a strict follow-up and offered potentially curative management for recurrent HCC.

## Data Availability

The datasets used and/or analyzed during the current study are available from the corresponding author on reasonable request.

## Abbreviations

HCC: Hepatocellular carcinoma
MC: Milan criteria
OS: Overall survival
RFS: Recurrence free survival
RTDS: Recurrence-to-death survival
SR: Surgical resection
RSR: Repeat surgical resection
RFA: Radiofrequency ablation
HR: Hazard ratio
CI: Confidential interval
HBV: Hepatitis B virus
MVI: Microvascular invasion
AFP: Alpha fetal protein
